# Risk Factors for Acute Respiratory Infection in the Australian Community

**DOI:** 10.1371/journal.pone.0101440

**Published:** 2014-07-17

**Authors:** Yingxi Chen, Emlyn Williams, Martyn Kirk

**Affiliations:** 1 National Centre for Epidemiology and Population Health, The Australian National University, Canberra, Australia; 2 Statistical Consulting Unit, The Australian National University, Canberra, Australia; Kliniken der Stadt Köln gGmbH, Germany

## Abstract

**Objectives:**

The objective of this study was to identify the risk factors for ARI in the Australian community.

**Methods:**

We used a national survey of 7578 randomly selected respondents in 2008–2009 to identify the risk factors of ARI. A case was defined as a person experiencing cold or flu with one or more symptoms of: fever, chills, sore throat, runny nose, or cough in the previous four weeks.

**Results:**

There were 19.8% (1505/7578) of respondents who reported ARI in the four weeks prior to the survey. Age was an independent risk factor for ARI, with the risk of acquiring ARI decreasing as age increased. Respondents reporting asthma (OR 1.4, 95%CI: 1.2–1.7) or having someone in their house attending childcare (OR 1.6, 95%CI: 1.2–2.1) were more likely to report ARI.

**Conclusions:**

It is important to identify ways of interrupting transmission of ARI amongst children. Improving identification of risk factors will enable targeted interventions for this exceedingly common syndrome.

## Introduction

Acute Respiratory infections (ARI) are a leading cause of morbidity and mortality globally, accounting for approximately 5.8 million deaths worldwide in 2010 [1]. In Australia, respiratory infections are estimated to result in six million general practitioners (GPs) consultations annually, and the combined death rate for pneumonia and influenza positions these diseases as the sixth leading cause of death-resulting 2715 deaths (2% of all deaths) in 2006–2007 [2], [3].

While antibiotics are effective against some bacterial infections, many respiratory infections are caused by range of other pathogens, such as viruses [4]. The diversity of micro-organisms causing ARI, and a diagnostic deficit of around 30%, make the formulation of an effective universal treatment for this disease impossible [5]. Therefore, the identification of risk factors for ARI in the community is important for developing effective policies and strategies to interrupt transmission and improve health outcomes. Studies in developing countries have reported that growth, nutritional factors, and parental smoking were associated with ARI [6]–[10]. However, due to the differences in both living condition and environmental circumstances, these results cannot be directly applied to industrialized countries, such as Australia.

Australian studies have identified that gender, maternal education, exposure to childcare, preschool, family income, and private health insurance coverage have an association with ARI amongst children [11]–[13]. However, the majority of the studies have been restricted to specific group (mainly children less than 5 years of age) and few studies have focused on the identification of risk factors for ARI in all age groups. Therefore, the risk factors for ARI in the Australian community remain poorly understood. Additionally, as published studies have been conducted in selected communities and have not been weighted to the population, standardized population data are not available, and the results may not be applicable to the general Australian population.

The aim of this study was to identify risk factors for ARI from a national cross-sectional survey to better understand the risk factors of ARI in the Australian community.

## Methods

As part of a national study assessing changes in the prevalence of gastroenteritis in Australia, a computer assisted telephone-based survey was administered to collect data regarding common illnesses over a one year period from 2008–2009. This study was a repeat of an earlier cross sectional survey [14]. The questionnaire covered information on gastrointestinal and respiratory symptoms, specifically cold or flu and associated fever or chill. In the survey, respondents were asked about potential environmental and demographic exposures potentially related to infection. In this paper, we present analyses regarding risk factors for respiratory symptoms.

### Case definition for analysis

Participants were asked if they had experienced an acute respiratory episode in the four weeks prior to the interview, which was defined as any episode of cold or flu, with at least one of the following symptoms: fever, chills, sore throat, running nose, or cough.

### Analysis

To identify the risk factors of ARI in the Australian community, survey data were post-stratified to adjust for known differences between the survey sample and the target population. We weighted the data to the 2008 resident population for age, sex and State from the Australian Bureau of Statistics (www.abs.gov.au). Logistic regression was used to produce crude odds ratios (ORs) separately for each of the geographic, demographic and socioeconomic variables. To do this, each predictor was treated as a categorical variable. We also used a generalized linear model with logit link to carry out the logistic regression over all the independent variables and obtain an analysis of deviance table. The significant variables were then further analyzed to investigate pairwise interactions. The Hosmer-Lemeshow test was used to check model fit. Design-based analyses were undertaken with the statistical package Stata version 12.1 using svy commands. Seasons were defined as follows: spring: September to November; summer: December to February; autumn: March to May; winter: June to August.

### Ethical considerations

As this was a national telephone-based survey, we were unable to obtain written consent from participants. During interview, oral consent was obtained from all participants and from parents and guardians on behalf of children. Verbal consent was recorded in an electronic database, along with other study data. The study and consent procedures were approved by ethics committees of the Australian Government Department of Health and Ageing, the Australian National University and the NSW Cancer Council.

## Results

Overall, of the 7578 study participants, 4554 participants (60.1%) were female, 249 (3.3%) were aged under five, and 6583 (87.8%) were >20 years old, and 161 (2.1%) were Aboriginal and Torres Strait Islander respondents.

### Risk factors for acute respiratory infections

Table 1 presents the results of separate logistic regressions for each independent variable. In the separate logistic models, significant associations were observed for age, Indigenous status, exposure to childcare, household size, household income, chronic diseases, asthma, and season. From the generalized linear model with all variables, analysis of the deviance table showed the significant predictor variables were age, exposure to childcare, asthma and season. Further analysis of these significant variables to include pairwise interactions showed no significant interactions (Table 2). The results of the final logistic regression model are presented in Table 3.

**Table 1:** Logistic regression examining the association between Acute Respiratory Infections and various risk factors, weighted for age, sex and State or Territory of residence, Australia 2008–9.

Risk factors		No of reporting		Weighted separate logistic regression	
		ARI (%)	No ARI (%)	OR (95%CI)	*p*
**Sex**	Female	917 (20.14)	3637 (79.86)	1.00 (def)	-
(reference = female)	Male	588 (19.44)	2436 (80.56)	0.84 (0.72–0.98)	0.023
**Age group** (years)	0–4	116 (46.59)	133 (53.41)	1.00 (def)	-
(reference = 0–4)	5–9	97 (38.96)	152 (61.04)	0.80 (53–1.22)	0.31
	10–19	187 (37.63)	310 (62.37)	0.65 (0.45–0.94)	0.02
	20–29	157 (30.97)	350 (69.03)	0.48 (0.33–0.70)	<0.001
	30–39	193 (24.06)	609 (75.94)	0.37 (0.26–0.53)	<0.001
	40–49	206 (19.36)	858 (80.64)	0.29 (0.20–0.41)	<0.001
	50–59	231 (16.81)	1143 (83.19)	0.21 (0.15–0.30)	<0.001
	60–64	109 (14.44)	646 (85.56)	0.20 (0.13–0.29)	<0.001
	+65	209 (10.04)	1872 (89.96)	0.12 (0.09–0.17)	<0.001
**Household size**	1	229 (13.19)	1507 (86.81)	1.00 (def)	-
(reference = 1)	2	423 (15.28)	2345 (84.72)	1.30 (1.02–1.66)	0.035
	3	267 (25.09)	797 (74.91)	2.65 (2.04–3.44)	<0.001
	4	350 (28.55)	876 (71.45)	2.87 (2.25–3.67)	<0.001
	5	147 (28.32)	372 (71.68)	2.70 (1.99–3.65)	<0.001
	≥6	87 (37.66)	144 (62.34)	3.92 (2.67–5.76)	<0.001
**Season**	Autumn	384 (21.02)	1443 (78.98)	1.00 (def)	-
(reference = Autumn)	Winter	550 (27.31)	1464 (72.69)	1.57 (1.28–1.93)	<0.001
	Spring	367 (19.53)	1512 (80.47)	0.98 (0.79–1.22)	0.876
	Summer	204 (10.98)	1654 (89.02)	0.49 (0.38–0.64)	<0.001
**Indigenous status**	Non-Indigenous	1459 (19.70)	5947 (80.30)	1.00 (def)	-
(reference = Non-indigenous)	Indigenous	46 (28.57)	115 (71.43)	1.98 (1.18–3.32)	0.01
**Childcare**	No	1328 (18.64)	5796 (81.36)	1.00 (def)	-
(reference = No)	Yes	174 (38.84)	274 (61.16)	2.31(1.79–2.97)	<0.001
**Healthcare card**	No	1102 (20.46)	4285 (79.54)	1.00 (def)	-
(reference = No)	Yes	373 (17.73)	1731 (82.27)	0.95 (0.79–1.14)	0.591
**Income ($AUD)**	<25000	215 (13.10)	1426 (86.90)	1.00 (def)	-
(reference = <25000)	25–50000	264 (18.87)	1135 (81.13)	1.79 (1.27–2.54)	<0.001
	50–100000	461 (22.78)	1563 (77.22)	1.72 (1.25–2.37)	<0.001
	≥100000	352 (24.24)	1100 (75.76)	2.23 (1.61–3.10)	<0.001
**Locality**	Majority city	844 (20.53)	3267 (79.47)	1.00 (def)	-
(reference = major city)	Town	464 (20.17)	1836(79.83)	0.91 (0.77–1.08)	0.30
	Rural community	178 (17.02)	868 (82.98)	0.62 (0.48–0.80)	<0.001
	Remote Australia	16 (17.39)	76 (83.61)	0.88 (0.35–2.17)	0.78
**Chronic disease**					
Asthma	No	1214 (18.87)	5277 (81.30)	1.00 (def)	-
	Yes	287 (26.72)	787 (73.28)	1.53 (1.26–1.87)	<0.001
Chronic lung disease	No	1475 (20.02)	5891 (79.98)	1.00 (def)	-
	Yes	25 (12.63)	173 (87.37)	0.63 (0.35–1.11)	0.108
Diabetes	No	1410 (20.17)	5581 (79.83)	1.00 (def)	-
	Yes	93 (16.20)	481 (83.80)	0.77 (0.56–1.07)	0.12
Spleen removed	No	1502 (19.89)	6051 (80.11)	1.00 (def)	-
	Yes	3 (12.00)	22 (88.00)	1.15 (0.30–4.43)	0.84
Heart disease	No	1439 (20.43)	5605 (79.57)	1.00 (def)	-
	Yes	64 (12.57)	445 (87.43)	0.45 (0.32–0.64)	<0.001
Arthritis	No	1236 (22.12)	4352 (77.88)	1.00 (def)	-
	Yes	265 (13.51)	1697 (86.49)	0.43 (0.35–0.52)	<0.001
Other lung disease	No	1464 (19.93)	5880 (80.07)	1.00 (def)	-
	Yes	38 (17.27)	182 (82.73)	0.86 (0.53–1.40)	0.54
Hypertension	No	1219 (21.64)	4414 (78.36)	1.00 (def)	-
	Yes	283 (14.67)	1646 (85.33)	0.52 (0.43–0.62)	<0.001
Cancer	No	1436 (20.19)	5676 (79.81)	1.00 (def)	-
	Yes	67 (14.60)	392 (85.40)	0.60 (0.43–0.83)	0.00

**Table 2:** Analysis of deviance table to investigate possible interactions between the significant predictor variables for Acute Respiratory Infections, Australia 2008–9.

Change	Model degrees of freedom	Deviance	Mean deviance	Deviance ratio	Approx F pr
+age	8	431.3420	53.9178	53.92	<0.001
+season	3	160.8938	53.6313	52.63	<0.001
+childcare	1	20.6574	20.6574	22.63	<0.001
+asthma	1	22.3245	22.3245	24.46	<0.001
+season.age	24	29.1504	1.2146	1.33	0.129
+season.childcare	3	1.6811	0.5604	0.61	0.606
+season.asthma	3	2.1597	0.7199	0.79	0.500
+age.childcare	8	3.8162	0.4770	0.52	0.840
+age.asthma	8	9.3464	1.1683	1.28	0.249
+asthma.childcare	1	0.9577	0.9577	1.05	0.306
Residual	7498	6844.32	0.9128	-	-
Total	7558	7526.6643	0.9959	-	-

**Table 3:** Results from the final logistic regression model showing the association between Acute Respiratory Infections and main predictors, data weighted for age, sex and State or Territory of residence, Australia 2008–9.

Risk factors		Logistic regression	
		OR (95%CI)	*p*
**Age group** (years)	0–4	1.00 (def)	**-**
(reference = 0–4)	5–9	0.83 (0.54–1.29)	0.41
	10–19	0.72 (0.49–1.06)	0.10
	20–29	0.56 (0.38–0.83)	<0.001
	30–39	0.40 (0.28–0.58)	<0.001
	40–49	0.33 (0.23–0.48)	<0.001
	50–59	0.25 (0.17–0.36)	<0.001
	60–64	0.24 (0.16–0.36)	<0.001
	+65	0.15 (0.10–0.21)	<0.001
**Season**	Autumn	1.00 (def)	-
(reference = Autumn)	Winter	1.63 (1.32–2.02)	<0.001
	Spring	1.03 (0.82–1.29)	0.84
	Summer	0.53 (0.41–0.69)	<0.001
**Childcare**	No	1.00 (def)	-
(reference = No)	Yes	1.60 (1.20–2.13)	<0.001
**Asthma**	No	1.00 (def)	-
(reference = Autumn)	Yes	1.41 (1.15–1.74)	<0.001

#### Age, sex and Indigenous status

**Figure 1:**
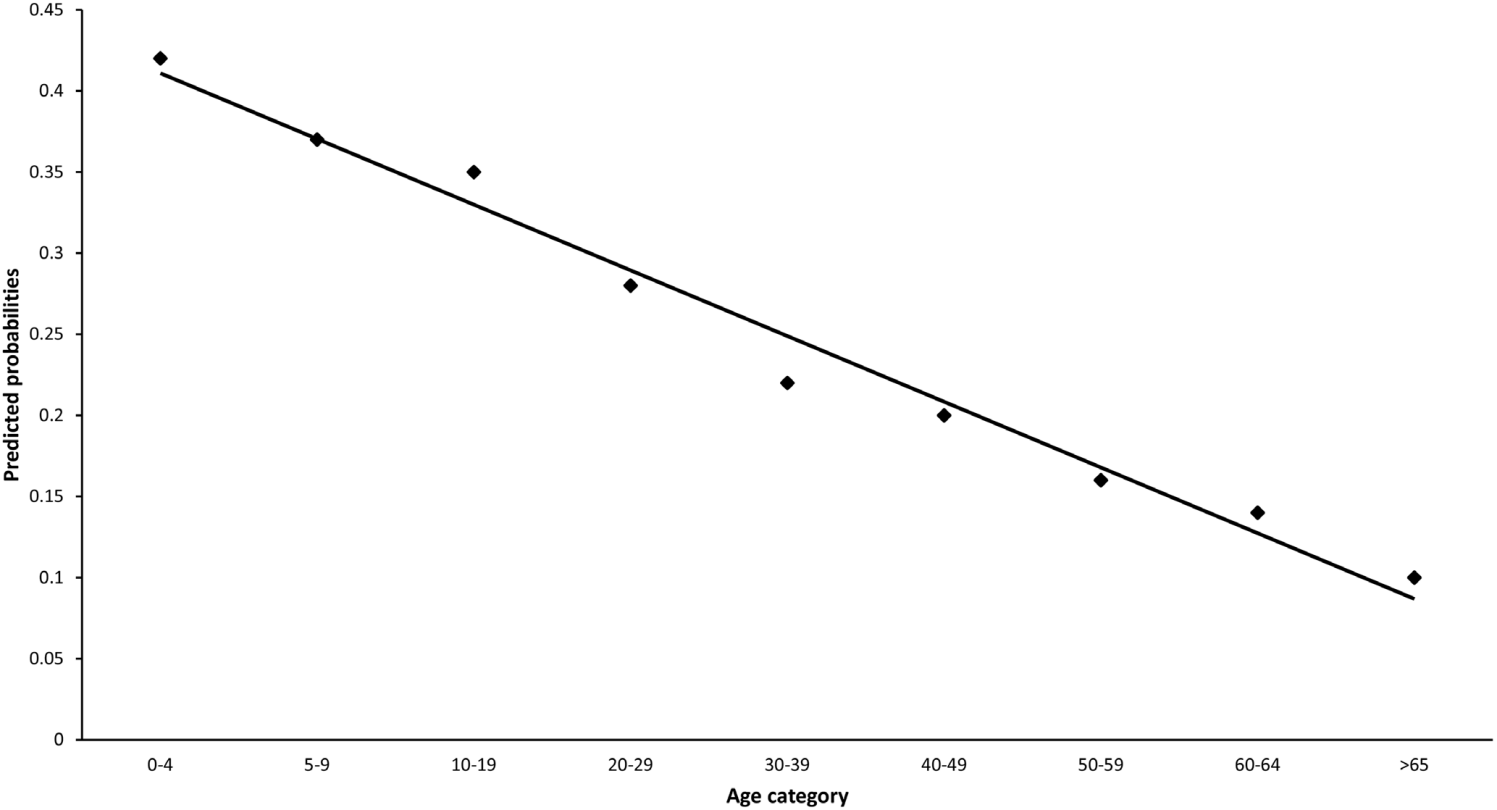
Predicted probabilities of each age group for Acute Respiratory Infections, weighted for age, sex and State or Territory of residence, Australia 2008–9.

A statistically significant association was identified for ARI across age groups both in the separate and final logistic regressions (P<0.001). Children 0–4 years of age were at the highest risk of acquiring ARI compared with other age groups, and odds ratios decreased as age increased. Additionally, we observed an almost linear relationship of the predicted probabilities of different age groups for ARI (Figure 1).

Gender was found to be associated with ARI in the separate logistic model, with males having a lower risk of experiencing respiratory infections compared to females (P = 0.023). Similarly, a significant difference was observed for Indigenous status in the separate logistic regression model (P = 0.009). However, in view of the small numbers of Indigenous respondents enrolled, Indigenous status was not a significant predictive variable for ARI, and was not included in the final logistic model.

#### Season

Season was an independent predictor for ARI. The final logistic model suggested a statistically significant difference in ARI across seasons (P<0.001), with a highest odds of ARI in winter (OR = 1.63) compared with autumn as the baseline, and the lowest odds in summer (OR = 0.53). This indicates that people were at the highest risk of acquiring ARI during winter, and less likely to experience ARI in summer. No significant difference was detected between spring and autumn.

#### Exposure to childcare

Having a child attending childcare in the household was found to be an independent risk factor for ARI after adjustment for other predictors in the final logistic model (P<0.001). Respondents having a family member attending childcare were at a higher risk of experiencing ARI compared to respondents without childcare exposure (OR = 1.60).

#### Chronic diseases

A marked association was identified between asthma and ARI (P<0.001) after controlling for potential confounding in the final logistic model, and respondents with asthma were more likely to experience ARI compared with respondents without asthma (OR = 1.41). Importantly, asthma was detected as an independent risk factor for ARI with no significant interactions between asthma and other main predictors.

Respondents with heart disease, arthritis, hypertension and cancer were found less likely to report ARI in the separate logistic model. However, this does not mean that these chronic conditions are protective factors for ARI; as age was strongly associated with chronic diseases, for example heart disease (data not shown), and age was also found to be a significant predictor for ARI, and therefore the detected associations between those diseases and ARI were confounded by age.

#### Household size and socioeconomic status

A significant difference in odds ratios was identified for different sized households in the separate logistic model (P<0.001). We observed dose response of increasing odds for increasing household size. Respondents from large households (>6) were at the highest risk of experiencing ARI compared with respondent from small households (reference = 1), with an OR of 3.92. People from rural communities were less likely to report ARI compared with people living in major cities (P<0.001).

There is a tendency for people with higher household incomes (over $AUD 100 000 per year) to have a higher risk of experiencing ARI in the separate logistic model. However, as this could be because children from higher income family are more likely to attend child-care as both parents work outside home [12], the result may be confounded by child-care attendance. Therefore, household income was not a main predictor for ARI.

While significant associations between ARI and household size and other indicators of socioeconomic status were detected in the separate logistic regressions, none of them were found to be statistically significant predictors for ARI when we used the generalized linear regression with logit link to measure the predictive role of each variable (data not shown). These variables were therefore not included in the final logistic regression model.

### Goodness-of-fit

The Hosmer-Lemeshow (GOF) test showed a good fit for the final logistic model (P = 0.97).

## Discussion

To our knowledge, this is the first nationally representative study investigating risk factors for ARI in the Australian community. Importantly, we found that age, season, having a family member attending childcare, and asthma were independently associated with ARI in the final logistic regression model. We have previously shown that ARI is one of the most common infectious diseases in the Australia population; resulting in approximately 68.9 million cases (95% CI 65.1–72.7) annually [15].

We found a significant inverse relationship between age and the risk of ARI. Children <5 years of age were at the most risk of acquiring ARI in Australia, which was similar to findings in other countries [16], [17]. It is likely that the behavior of young children, for instance lack of awareness of hand hygiene or other hygiene issues, may increase their exposure to pathogens via person-to-person contact. Nevertheless, asymptomatic individuals may shed virus, and therefore be infectious to others [18]. Studies have reported that children can shed respiratory viruses, such as influenza viruses, for longer periods than adults, thus increasing the exposure to pathogens for others [19]. Additionally, young children may be more likely to experience infectious diseases due to the immaturity of their immune system [10], [20].

Similarly, the lower risk of adult respondents to ARI may be a consequence of the maturity of their immune system or natural immunity obtained through repeated infections of bacteria and viruses [21]. People over 65 years old have a distinctly lower risk of experiencing ARI. This may be due to social isolation and therefore having minimal contact with infected individuals, primarily children. Additionally, a certain degree of prior natural immunity to influenza has been recognized in the Australian population, particularly in those over 60 years of age [22]. This, along with prior immunity to other respiratory viral pathogens, could also explain their reduced risk of acquiring ARI.

Seasonally, we observed the highest odds ratio of ARI in winter compared with other seasons, suggesting that climatic variation may influence the transmission of ARI in Australia. Research on the environmental effects of respiratory infection transmission has found a connection between the temperature and air humidity and the transmission of certain types of viruses. Routine surveillance of Influenza Like Illness (ILI) has also shown increased incidence during colder months [23], [24]. A recent study also found that vasoconstriction of the upper respiratory system due to chilling of the feet in cold water may encourage the onset of common cold symptoms [25].

While the effects of weather and climate on disease transmission have been widely investigated, some researchers have highlighted the important role of population immunity and overall susceptibility to infections as the mechanisms responsible for the seasonality of respiratory infections. Previous studies have identified that deficiency in vitamin D impairs the body’s antimicrobial system [26]. Therefore, asymptomatic infectious individuals may become contagious during winter due to reduction in exposure to ultra-violet radiation [27].

Having family members attending childcare facilities was found to be a strong risk factor for acute respiratory illness in both separate and final logistic regression. Previous studies have consistently found that children attending childcare centers are at a higher risk of experiencing ARI, compared to children in home care, due to the physical environment in those facilities and the transmission via person to person contact [28], [29]. However, the association between having a family member attending child-care and ARI has not been widely explored. Our study suggested that having a family member attending childcare was strongly associated with acute respiratory infections, and several contributing factors may explain the detected association.

One possible explanation could be the transmission of disease via person-to-person contact amongst family members [30]. As children attending child-care are at higher risk of experiencing ARI, and therefore family members are more likely to be infected through contact with an infected child. Additionally, people having children attending child-care are more likely to live in a larger household (data not shown). Given that households have a significant influence on the transmission of respiratory infections, as households become larger, the number of potential interactions between infectious and susceptible individuals increases [31], people with family members at child-care are at higher risk of experiencing ARI. Therefore, interrupting the transmission of infections within households is important in reducing secondary attack rate. Previous studies have shown that many rhinovirus infections in adults are asymptomatic, and are often acquired from symptomatic children in the family [32]. This suggests that efforts to prevent respiratory virus transmission within households should focus on symptomatic young children.

As children attending child-care were at higher risk of acquiring respiratory infections than those in home-care [33], several possible strategies for childcare facilities might help to reduce acute respiratory infections episodes amongst children and also their families. While the excess risk of ARI associated with childcare is highest in children under one years old [34], previous infection control studies have found that a high compliance with infection control practices, such as hand-washing and aseptic nose-wiping technique could help to reduce the direct transmission of respiratory infections amongst this age group in child-care [35]. Also, identifying environmental determinants of infection control behavior and adequate isolation policies to interrupt the transmission between the sick children and other could also help to reduce the childcare associated respiratory infection episodes [36].

Our study found also that asthma was an independent risk factor of ARI after adjusting for age, season and childcare exposure. Recent observational studies have shown a similar association between respiratory infections and asthma exacerbations, and lower respiratory infections has been identified as independent risk factors for asthma [37], [38]. In a study of children, acute asthma episodes were substantially increased during respiratory syncytial virus (RSV) episodes [39]. This is probably because certain types of respiratory viruses help to produce more interleukin-11 (IL-11), which exacerbates asthma from pulmonary stromal cells [40]. Other viruses, such as human rhinovirus, are also commonly associated with asthma exacerbations in older children [41], [42]. Children who experienced ARI secondary to rhinovirus infection are at particular risk due to underlying respiratory conditions such as asthma [43]. However, the relationship between respiratory infections and the induction of asthma is complex and may also involve interactions between host factors and the number and severity of infections.

## Conclusion

We observed that risk factors, such as young age, season, childcare exposure and asthma, were significantly associated with ARI. The improved identification of risk factors is useful to guide interventions to minimize transmission and improve public health resource allocation. Age was strongly associated with ARI, with young children aged five years or less significantly more likely to report respiratory episodes. Households having a family member attending childcare were clearly at higher risk of ARI. Measures can be implemented to reduce the high risk of children acquiring infections at childcare (including the promotion of hygiene and the age composition of the children in attendance [44]), and ultimately reduce the transmission from contagious children to other household members. There is also scope to identify and promote simple measures to interrupt the transmission of infections between the sick and their family members within households. In our study, asthma was identified as an independent risk factor for ARI and further analysis is needed to provide the comprehensive understanding of the causal relationship between asthma and respiratory infections.
